# The histological characteristics, age-related thickness change of skin, and expression of the HSPs in the skin during hair cycle in yak (*Bos grunniens*)

**DOI:** 10.1371/journal.pone.0176451

**Published:** 2017-05-02

**Authors:** Xue Yang, Yan Cui, Jing Yue, Honghong He, Chuan Yu, Penggang Liu, Jun Liu, Xiandong Ren, Yun Meng

**Affiliations:** College of Veterinary Medicine, Gansu Agricultural University, Lanzhou, Gansu, China; University of Kansas School of Medicine, UNITED STATES

## Abstract

**Objective:**

This experiment was conducted to study the histological characteristics, age-related thickness changes, and expression of HSPs in the skin of yak.

**Methods:**

A total of 20 yaks (10 males and 10 females) were used. Different regions of the normal skin of three different ages (newborn, half-year-old and adult) of yaks were harvested for histological study and thickness measurement. Biopsy samples were taken from the scapula regions of the skin from the same five approximately 1-year-old yaks during the hair cycle (telogen, anagen and catagen). RT-PCR, western blot and immunohistochemistry methods using the mRNA and protein levels were used to detect the expression of HSP27, HSP70 and HSP90. RT-PCR method was used to detect the mRNA expression of CGI-58 and KDF1. The IPP6.0 software was used to analyze the immunohistochemistry and measure the thickness of the skin.

**Results:**

The general histological structure of hairy yak skin was similar to other domestic mammals. The unique features included prominent cutaneous vascular plexuses, underdeveloped sweat glands, a large number of nasolabial glands in the nasolabial plate, and hair follicle groups composed of one primary follicle and several secondary follicles. The skin, epidermis and dermis thickness did vary significantly between different body regions and different ages. The thickness of the skin, epidermis and dermis increased from newborn to adult in yaks. Yak skin thickness decreased from dorsally to ventrally on the trunk. The skin on the lateral surface was thicker than the skin on the medial surface on the limbs. HSP27, HSP70 and HSP90 showed different expression patterns during the hair cycle using RT-PCR, western blot and immunohistochemistry methods. The expression of HSP27 mRNA and protein in the anagen stage was the highest, followed by the catagen stage, and the expression in the telogen stage was the lowest. The expression of HSP70 mRNA and protein in the telogen stage was the highest, followed by the anagen stage, and the expression in the catagen stage was the lowest. The expression of HSP90 mRNA and protein in the anagen stage was the highest, followed by the telogen stage, and the expression in the catagen stage was the lowest. HSPs were mainly expressed in the outer root sheath of hair follicle during the hair cycle, also expressed in epidermis, sebaceous gland and sweat gland in the skin of Yak. The expression of CGI-58 mRNA in the anagen stage was the highest, followed by the catagen stage, and the expression in the telogen stage was the lowest. The expression of KDF1 mRNA in the telogen stage was the highest, followed by the catagen stage, and the expression in the anagen stage was the lowest.

**Meaning:**

In this study, we examined and fully described the histology of normal skin in Yak and measured the skin thickness of different ages and different regions in Yak. These data may be useful to better understand and appreciate the adaptability features of yak skin. Our investigation reports the expression patterns of HSPs in yak skin for the first time. The different expression pattern of HSPs during the hair cycle suggests they may play different roles in yak hair follicle biology.

## Introduction

Yak (*Bos grunniens*) is a special plateau mammal that lives in the extreme environments of the Tibetan highlands, which has the basic features of extreme cold, high altitudes with reduced oxygen content in the air, and high ultraviolet radiation. The altitude where yaks live normally is over 3000–6000 meters high. The annual temperature of this area is -3 to 3°C, and the extreme lowest temperature is -40°C. Yaks play an important role in normal life for people living in the plateau, such as providing meat, milk and wool, packing goods and materials and riding.

As a special plateau mammal, there are many studies on their reproductive performance, including reproductive organ structures [1–4], hormone regulation [5–8] and adaptability. Studies on the adaptability of yak are mostly concentrated on the respiratory system [9–11], circulatory system [12–14] and immune system [15,16], including the histological structure, ultrastructure and distribution of some factors such as VEGF, HIF and CX43.

The skin is the largest organ of the body and serves many functions, such as protection against environmental aggressions (cold, intense radiation and sandstorms), sensation, metabolism and thermoregulation. There are some studies of the skin histology of some mammals such as llamas [17], sheep [18], ferrets [19] and camels (Camelus dromedaries) [20]. It is an accepted fact that skin varies considerably in thickness based on its site and age. In 2009, Volkering measured the skin thickness over the equine body [21]. Many of the related works that have been conducted on different body regions and ages have been done in humans [22–24]. Only a few studies have been published on the histological research and measurement of yak skin, but there are no detailed data for skin histologic characteristics, thickness changes or the relationship between structure and adaptability in yak.

Heat shock protein (HSP) is one type of molecular chaperone and includes five major groups: 20–30, 60, 70, 90 and 110 kDa based on molecular size [25]. HSPs are involved in protein folding, assembly, transport and regulation of cell growth and differentiation [26]. Heat shock protein-27 (HSP27) is a member of the small heat shock proteins (sHSP). The primary structure of HSP27 is highly homologous to other members of the sHSP family; it contains the conserved α-crystallin domain and differs in the C- and N-terminal regions. HSP27 is expressed in all human tissues, including astrocytes and primary neuronal cells, but is mainly found in skeletal, smooth and cardiac muscles [27]. HSP27 protein is expressed in a differentiation-related pattern [28–32]. For example, keratinocytes of the upper epidermis express higher levels of HSP27 than basal cell keratinocytes in normal human skin. In developing human skin, HSP27 protein expression correlates with increasing epidermal differentiation and trichilemmal keratinization [33].

HSP70 included two major proteins: constitutively expressed HSC70 and stress-inducible HSP72 [34]. HSC70 is expressed in practically all organs and tissues and functions as ATP-dependent molecular chaperone under normal conditions [35–37]. HSP70 plays an important role in cell apoptosis through its ability to inhibit apoptosis. HSP70 can regulate cell apoptosis at different levels such as affecting some transcription factors involved in the expression of Bcl-2 family [38].

HSP90 belongs to another important HSPs family. It is a kind of abundant protein expressed in all eukaryotic calls [39,40]. HSP90 is highly conserved and also is an ATP-dependent chaperone. HSP90 can maturate, stabilize and activate a range of client proteins through form complex [40,41]. Many of these client proteins are involved in cell growth, proliferation and survival.

Hair follicle (HF) cycling transitions include telagen, anagen and catagen. These phases are controlled by molecular switches such as HSPs. To date, nothing is known about HSPs protein expressions in the skin of yaks or their relation to the cycling changes in HF. In this investigation, we tested the expression patterns of HSP27, HSP70 and HSP90 in normal yak skin during their hair cycle.

## Materials and methods

### Experimental animals and treatments

A total of 20 yaks (10 males and 10 females) from the Gannan Tibetan Autonomous Prefecture in Gansu province and Xining City in Qinghai province that were humanely euthanized for reasons unrelated to the skin were used in this study. Yaks were purchased from Jianguo Ma and Ming Liu, the small holders in Gannan Tibetan Autonomous Prefecture of Gansu Province and Datong County of Qinghai Province (China). Yaks were permitted as experimental animals by the owners. In order to maintain the original habitat, the yaks were executed and samples were collected in the local instead of being housed at the university. All of the yaks were in good nutritional condition and were distributed evenly into four groups (newborn, half-year-old, 1-year-old and adult) (Table 1). In this study, the experimental animals were all handled according to the Animal Ethics Procedures and Guidelines of the People’s Republic of China, and the study was approved by the Animal Ethics Committee of Gansu Agricultural University.

**Table 1:** Specimens examined.

Number	Age	Origin	Used for
5	Newborn	Gannan, Gansu	Measurement
5	half-year-old	Xining, Qinghai	Measurement
5	1-year-old	Xining, Qinghai	RT-PCR, WB, Immunohistochemistry
5	3–5 years	Xining, Qinghai	Histology, Measurement

**Fig 1:**
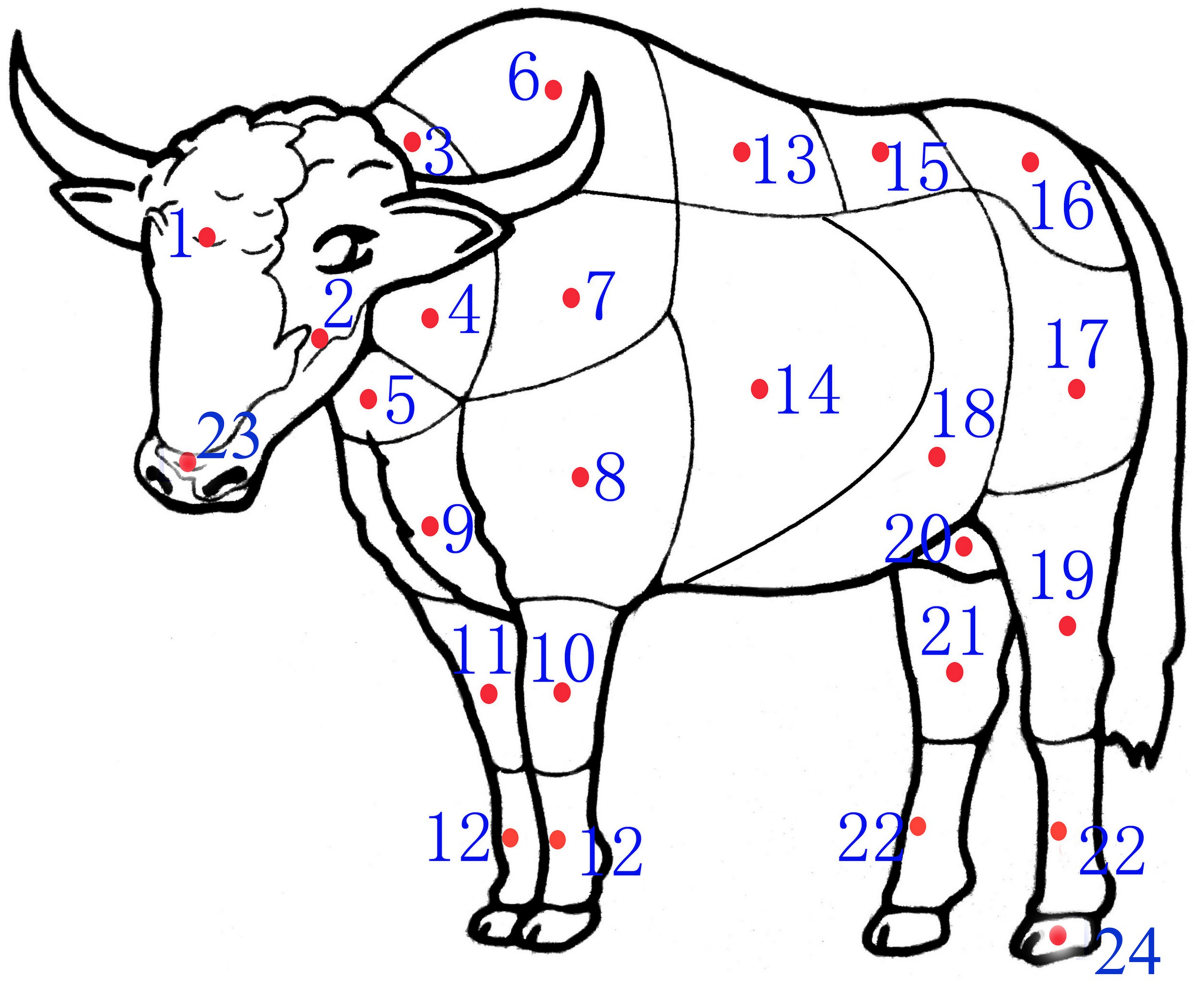
Regions of yak body where skin specimens were obtained for this study. 1 forehead 2 cheek 3 dorsal neck 4 lateral neck 5 ventral neck 6 withers 7 scapula 8 brachia 9 thorax 10 lateral of forearm 11 medial of forearm 12 metacarpus 13 back 14 costal region 15 waist 16 buttock 17 thigh 18 abdomen 19 lateral of crus 20 inguinal region 21 medial of crus 22 metatarsus 23 nasolabial plate 24 hoof 25 axilla. Note: the axilla was not marked.

Twenty-five different regions (Fig 1) of the normal skin of three different age (newborn, half-year-old and adult) yaks were harvested for histological study and thickness measurements. All of the animals were euthanized by intravenous injection of pentobarbital sodium (150 mg/kg body weight) for animal welfare and safety of experimenter.

Five of the 1-year-old yaks that were used for the hair follicle study were kept under the same natural photoperiod and temperature conditions. Biopsy samples of the skin during the hair cycle (telogen, anagen and catagen) were taken from the scapula region. Skin specimens used for immunohistochemistry were stored in 4% paraformaldehyde solution, and skin specimens used for RT-PCR and WB were stored at -80°C.

### Light microscopy

Skin samples from the yaks were fixed on the paperboard to prevent shrinkage, stored in 4% paraformaldehyde solution, softened, dehydrated, embedded in paraffin, sectioned at a thickness of 6 μm and deparaffinized. The sections were stained using hematoxylin and eosin (H.E), Masson’s trichrome, Weigert-van Gieson (WVG), Alcian blue periodic acid schiff (AB-PAS) and Sacpic [42] methods.

### Relative real-time RT-PCR

Total skin tissue RNA was isolated using TRIzol reagent (Invitrogen, CA, USA). RNA was reverse transcribed to single-strand cDNA using a Revertaid First Strand cDNA Synthesis kit (MBI Fermentas, Canada) according to the manufacturer's protocol. Reverse transcription was carried out using a PCR kit (Roche, Basel, Switzerland) in a 20 μL reaction containing 2 μg RNA, 50 mM KCl, 50 mM Tris/HCl, 4 mM MgCl_2_ and 10 mM of dNTPs, oligo-(dT) primers, RNAse inhibitor and MuLV reverse transcriptase. The reaction mixture was incubated for 5 min at 37°C, 60 min at 42°C, and then heated to 70°C for 5 min in a thermocycler (MBI Fermentas, Canada). Quantitative real-time PCR was conducted with a PTC 200 real-time PCR reactor (MJ Research, Fremont, CA, USA) for SYBR green PCR master mix (Takara, Shiga, Japan) according to the manufacturer's protocol. The primers were designed according to the respective gene sequences using the Primer 3 software and were synthesized by Sangon Biotech (China). The PCR primers are shown in Table 2. The PCR conditions were 95°C for 30 s, 95°C for 4 s, 60°C for 1 min and 72°C for 30 s for a total of 42 cycles, with a final extension for 10 min at 72°C. The amplified PCR products were electrophoresed on a 1.5% agarose gel. Relative gene expression quantifications were quantified using Image-QuanT software (Molecular Dynamics, Sunnyvale, CA, USA) and calculated using the comparative Ct method with β-actin as an internal standard. In all cases, each PCR trail was performed with triplicate samples and repeated at least three times.

**Table 2:** Primers used in this study.

Genes	Primer sequences (5’-3’)	Length(bp)	Annealing(°C)
HSP27	F:CAGTGCGATACGAGCAGGAA	182	60
	R:CAGGACTTGGAAGCGGGAT		
HSP70	F:GCTGAACCCGCAGAACACG	158	58
	R:GCCTTGGTCTCCCCTTTGTAG		
HSP90	F:CAAGCAAGATCGAACCCTCAC	174	62
	R:GCTGAATAAAACCCGACACCA		
CGI-58	F: CATCCAGGGTTAGTCATCTC	189	52
	R: GCCTTAAACGCTGTACTAGAC		
	F: ACTCCACCCCCATAACACGC	165	62
KDF1	R: GGCACTGTCCACAGAGTTCCAGA		
β-actin	F:AGGCTGTGCTGTCCCTGTATG	207	62
	R:GCTCGGCTGTGGTGGTAAA		

### Western blot detection of HSPs

Total protein was extracted from skin tissues using radioimmunoprecipitation assay lysis buffer and was quantified using the Enhanced BCA protein assay kit (Bio Tek, VT, USA). Briefly, proteins were denatured at 100°C for 5 min and electrophoretically separated on a 10% gel using sodium dodecyl sulfate polyacrylamide gel electrophoresis (SDS-PAGE). Proteins were transferred onto polyvinylidene fluoride (PVF) membranes, and the membranes were blocked with 5% skim milk powder in Tris-buffered saline containing 0.1% Tween 20 (TBST) at RT for 30 min. The membranes were then incubated with monoclonal anti-HSP27 antibody (Abcam, Mouse ab79868, 1:1000 dilution), polyclonal anti-HSP70 antibody (Abcam, Rabbit ab79852, 1:1000 dilution) and monoclonal anti-HSP90 antibody (Abcam, Mouse ab13492, 1:1000 dilution) at 4°C overnight, respectively. On the following morning, the membranes were incubated with a horseradish peroxidase-conjugated goat anti-mouse IgM whole serum antibody (Bioss, Beijing, bs-0368Gs, anti-mouse, 1:2000 dilution) and goat anti-rabbit IgM antibody (Bioss, Beijing, bs-0295G-HRP, anti-rabbit, 1:2000 dilution). The internal loading control was β-actin. The expression of HSP27 protein was measured using chemiluminescence.

### HSPs immunohistochemical staining

Sections were labeled for HSP27 (Abcam, Mouse ab79868, 1:200 dilution), HSP70 (Abcam, Rabbit ab79852, 1:200 dilution) and HSP90 (Abcam, Mouse ab13492, 1:200 dilution) using the streptavidin/peroxidase complex immunostaining technique, respectively. Primary antibodies were incubated for 2 hours at 37°C. The reaction products were formed with diaminobenzidine. Nuclear counterstaining was performed with hematoxylin. Negative controls were obtained by omitting the first-layer antibody.

### Measurement and data analysis

Skin thickness was measured using a micrometer. The thickness of the epidermis and dermis was measured using a computerized light microscope (Olympus DP71) and morphometric software (Image-Pro plus 6.0). The epidermis was measured from the free margin of skin to the dermis papillae and epidermis ridge. The dermis was measured in the same way from the epidermis ridge and dermis papillae to the dermal-fat junction [24]. Data were expressed as the mean ± standard deviation (SD). Statistical analysis was performed using the Statistical Package for Social Science software, version 19.0 (SPSS Inc., Chicago, IL, USA). Statistical analysis was primarily conducted using a one-way analysis of variance (ANOVA). A *P* value of *P*<0.05 was considered statistically significant.

## Results

### Histologic characteristics of skin structure

#### Epidermis

**Fig 2:**
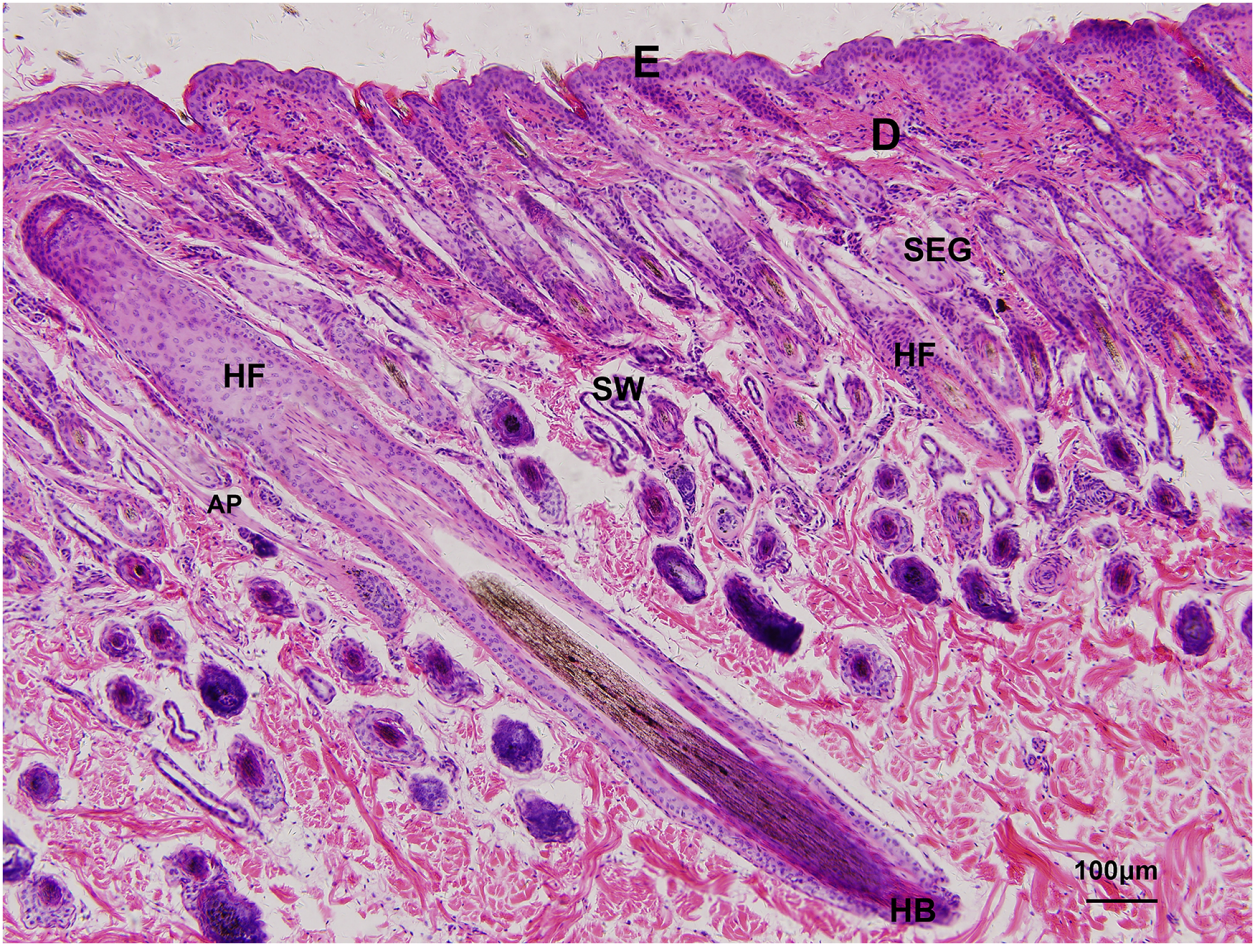
Histological characteristics of skin in yak. E: epidermis, D: dermis, SEG: sebaceous gland, SW: sweat gland, AP: arrector pili, HF: hair follicle, HB: hair bulb.

There was no obvious epidermal interpapillary peg in yak skin, but the epidermis appeared undulating from the epidermis of the opening in the hair follicle down to the dermis (Fig 2). The epidermis of hairy skin in yak consisted of four layers: stratum corneum, stratum granulosum, stratum spinosum and stratum basale, whereas the glabrous skin also included stratum lucidum, such as the nasolabial plate and the hooves.

**Fig 3:**
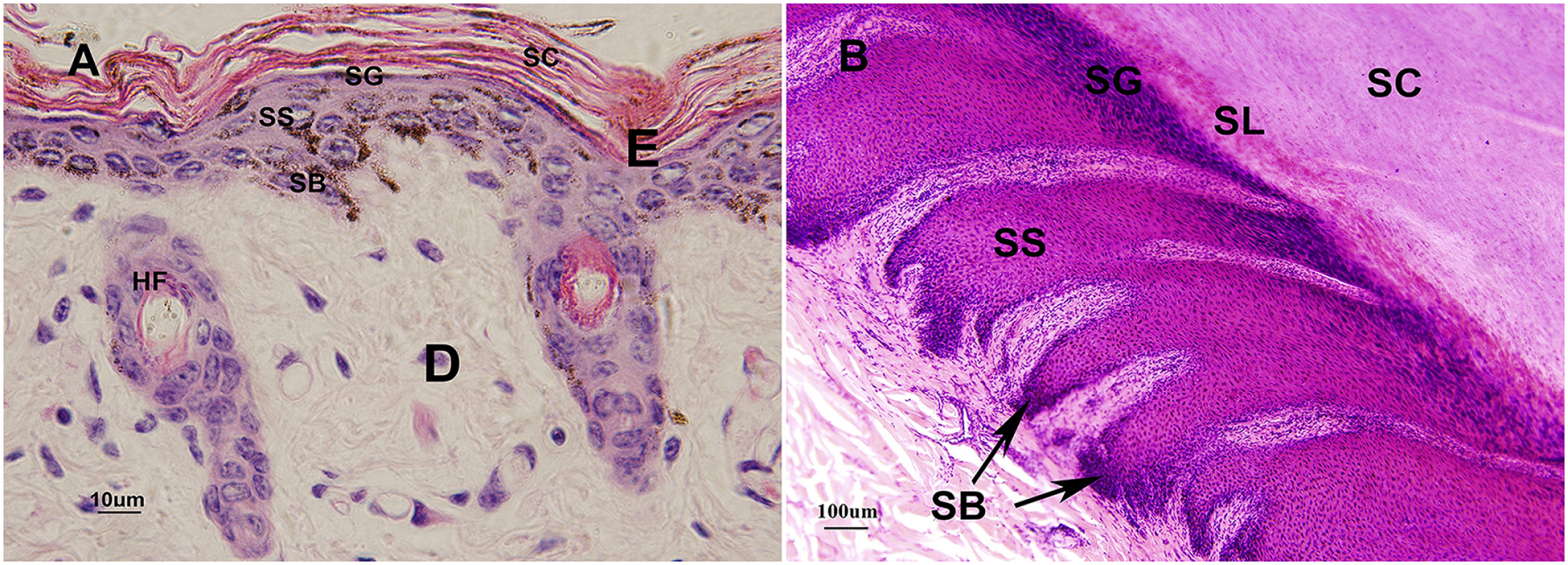
Histological structures of the epidermis in yak. A. Structures of epidermis in hairy skin of yak, HE ×1000 B. Structures of epidermis in glabrous skin of yak, HE ×400. SC: stratum corneum, SG: stratum granulosum, SS: stratum spinosum, SB: stratum basale, SL: stratum lucidum.

The stratum corneum was made of flattened, anucleated, scale-like cells that were fibrous and could easily fall off. The stratum lucidum was a pink uniform band that was found in the planum nasolabiale and was hoof coronal and hoof sphere. The stratum granulosum consisted of one layer of flattened cells, which would be keratinized into the corneum. The depth of the cell layer in the stratum spinosum was varied by age changing. In newborn yak, there were only 1–2 layers, although it increased to 3–4 layers in the half-year-old and adult yak. The stratum basale was made of cubical cells with large nuclei lying perpendicular to the basical membrane (Fig 3A and 3B).

#### Dermis

The dermis contained the papillary, which was in contact with the epidermis, and reticular layers, which were in contact with the underlying hypodermis. The dermis consisted of numerous fiber types and few cell types and contained numerous blood and lymphatic vessels, nerves, arrector pili, sweat glands, sebaceous glands, hair and hair follicles (Fig 2).

**Fig 4:**
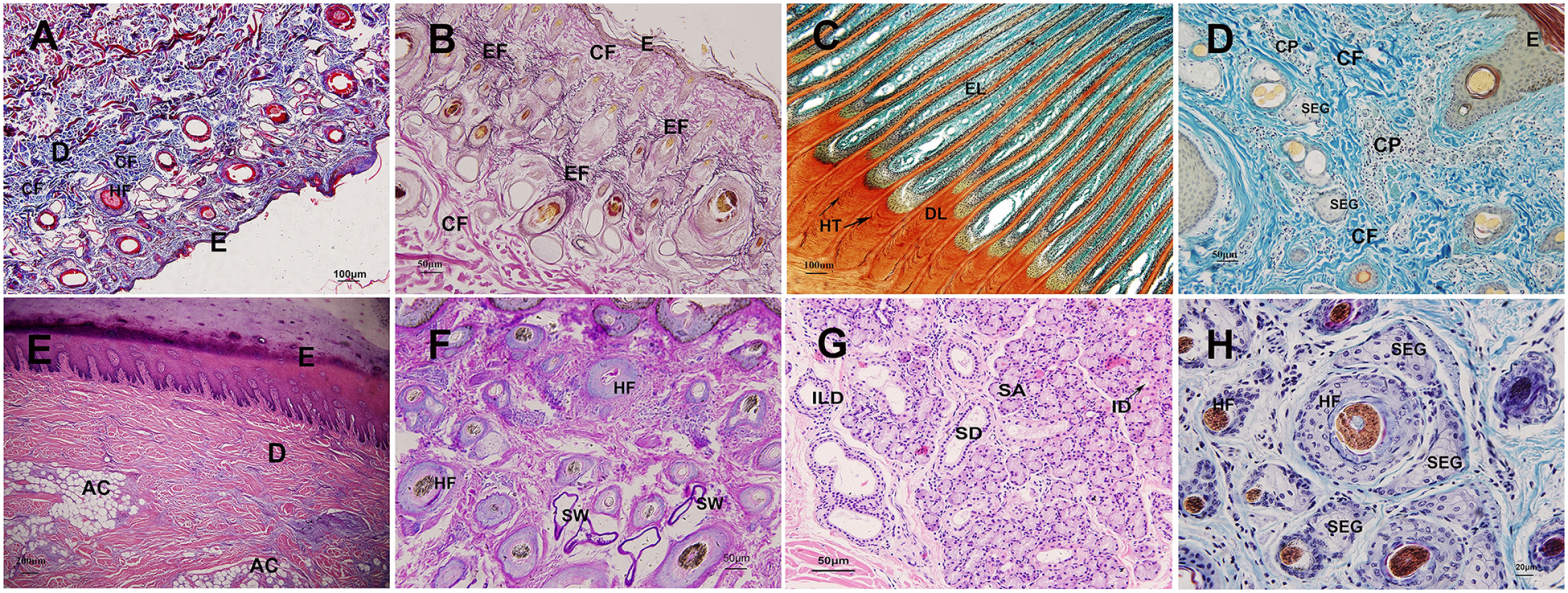
Histological structures of the dermis in yak. A. Collagen fiber in dermis in yak, Masson’s trichrome ×100 B. Elastic fiber in dermis in yak, WVG ×200 C. Epidermal lamellae and dermal lamellae fit together in hoof in yak, Sacpic ×100 D. Capillary plexus in dermis in yak, Sacpic ×200 E. Adipose tissue in the deep dermis of hoof sphere, HE×40 F. Sweat gland and secretion in deep dermis in yak, AB-PAS ×200 G. Nasolabial glands distributed in the dermis of nasolabial plate, HE ×200 H. Sebaceous gland around the hair follicle in dermis, HE ×400 E: epidermis, D: dermis, CF: collagen fiber, EF: elastic fiber, HF: hair follicle, CP: capillary plexus, EL: epidermal lamellae, DL: dermal lamellae, HT: horn tubule, AC: adipose cell, SW: sweat gland, SD: striated duct, SA: serous alveoli, ID: intercalated duct, ILD: interlobular duct, SEG: sebaceous gland.

The dermis contained a variable amount of collagen and a few elastic and reticular fibers, which often were intertwined with each other (Fig 4A and 4B). The collagen fibers were arranged in bundles and appeared blue-green using Masson’s trichrome staining (Fig 4A). Compared with the papillary layer, collagen fiber bundles were thicker in the reticular layer. Elastic fibers were primarily located in the papillary dermis and surrounding vessels, which appeared black-green when using WVG staining (Fig 4B). In the hoof, the epidermal lamellae and the dermal lamellae fit together to form a tight junction (Fig 4C). A large number of capillary plexuses was distributed around the boundary between the papillary and reticular layers (Fig 4D). The arrector pili was a thin smooth muscle bundle that was located in the papillary layer and extended up to the epidermis (Fig 2). There was also some adipose tissue in the deep dermis in some body regions such as the hoof sphere (Fig 4E). In yak, few sweat glands were located in the deep dermis, and the secretion was blue-violet by AB-PAS staining (Fig 4F), which meant that the secretion was an acidic-neutral complex that contained many saccharides. Many nasolabial glands were distributed in the dermis of the nasolabial plate, which belonged to the branched tubuloacinar gland (Fig 4G). The sebaceous glands were well developed and distributed around the hair follicles (Fig 4H).

#### Hair follicle

**Fig 5:**
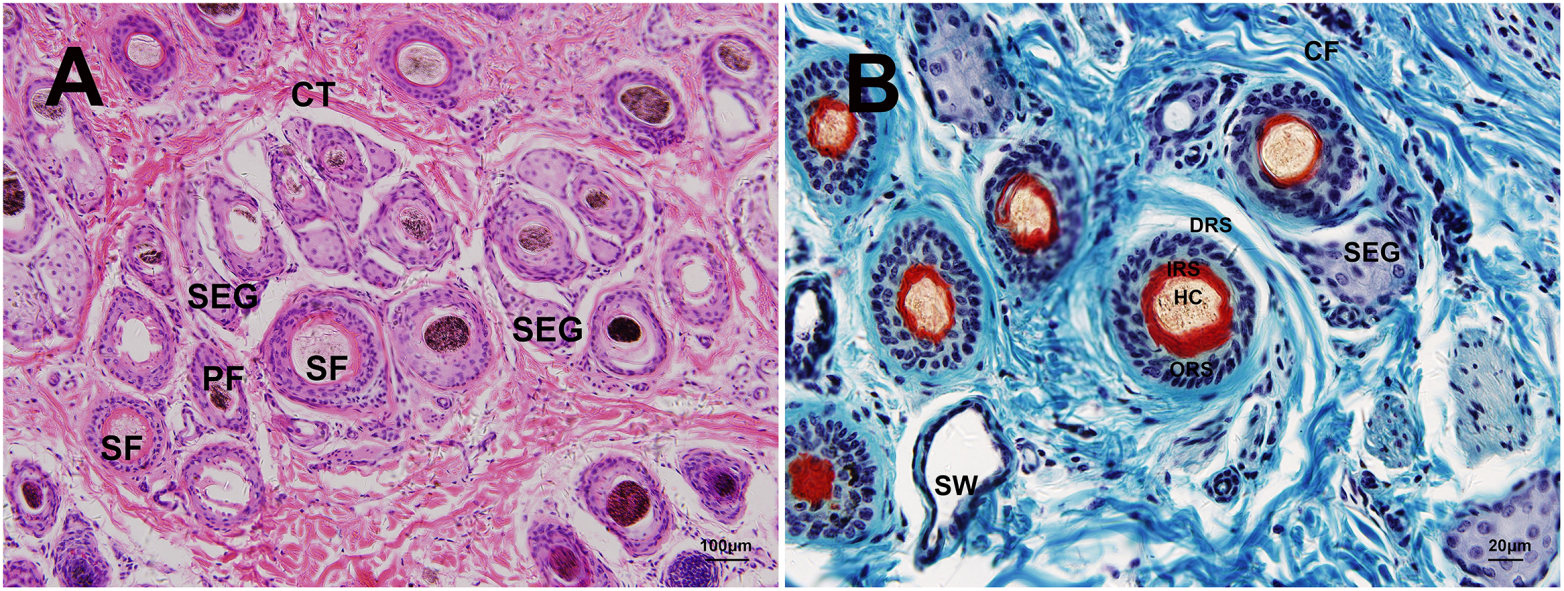
Histological structures of hair follicle in yak. A. Hair follicle group consisting of one primary follicle and some secondary follicles and sebaceous gland, HE ×100 B. Structures of hair follicle in yak, Sacpic ×400 SEG: sebaceous gland, SW: sweat gland, HF: hair follicle, CF: collagen fiber, CT: connective tissue, PF: primary follicle, SF: secondary follicle, DRS: dermal root sheath, ORS: outer root sheath, IRS: inner root sheath, HC: hair cortex.

The yak hair was longer and denser than other cattle breeds, and the thickness was different, with hair follicles that were variable in size, were evenly distributed in the papillary and upper reticular layers and often formed in groups. One hair follicle group consisted of one primary follicle (PF) and several secondary follicles (SF), which were accompanied by sebaceous glands. The hair follicle group was surrounded by a connective tissue sheath (CTS) (Fig 5A). The hair follicle was composed of the dermal root sheath (DRS), outer root sheath (ORS) and inner root sheath (IRS) (Fig 5B). The dermal root sheath was made of connective tissue. The outer root sheath possessed 3–4 layers, whereas the inner root sheath was composed of three layers: Henle's layer, Huxley's layer, and an internal cuticle. Specifically, Henle’s layer consisted of one single layer of cubical cells with flattened nuclei, and Huxley’s layer was composed of a cell layer with flattened nuclei. By contrast, the internal cuticle had one cell layer, which had been keratinized.

### Measurement of skin thickness

In the newborn group, the skin thickness ranged from 624 to 1538 μm; the inguinal region was thinnest (624 μm), and the back and cheek were the thickest (1500–1538 μm) (Tables 3 and 4). The thickness of the epidermis varied from 16.073 to 29.307 μm. The thickness in the lateral crus, cheek and inguinal regions ranged from 16.073 to 17.104 μm. The back, dorsal neck and forehead were relatively thick (21.913–23.584 μm), and the metacarpus was thickest (29.307 μm) (Tables 3 and 4). The thickness of the dermis varied from 389.956 to 948.520 μm, and that in the metatarsus, waist, axilla, lateral crus and the inguinal region ranged from 389.956 to 457.078 μm. The abdomen, dorsal neck and buttock were relatively thick (545.228–683.58 μm), and the back was thickest (948.520 μm) (Tables 3 and 4).

**Table 3:** Thickness of skin of body regions.

Region	Epidermis(μm)			Dermis(μm)			Skin(μm)			E/ (E+D) (%)		
	newborn	half-year-old	adult	newborn	half-year-old	adult	newborn	half-year-old	adult	newborn	half-year-old	adult
Forehead	23.584±3.770	28.259±5.909	46.248±9.211	890.758±70.745	1035.519±83.844	1805.675±120.657	1442±22.706	2188±34.254	2910±19.692	2.579	2.656	2.497
Cheek	16.714±3.389	29.050±4.969	39.114±5.116	715.009±71.733	721.014±70.315	1428.367±113.882	1538±33.267	2264±26.331	4640±13.032	2.284	3.873	2.665
Dorsal neck	22.564±5.543	34.372±6.243	44.296±8.768	651.727±109.538	1117.065±98.644	2003.075±223.575	1238±25.733	2364±22.706	4396±96.517	3.346	2.985	2.164
Lateral neck	21.718±4.034	30.406±5.498	57.781±13.830	668.446±67.268	687.223±80.938	1854.609±97.370	1226±32.728	2116±30.984	3946±109.158	3.147	4.237	3.021
Ventral neck	19.958±3.411	26.830±6.317	41.214±6.264	586.189±68.503	897.889±95.699	1969.327±150.976	1306±31.340	2276±30.984	4294±69.314	3.293	2.901	2.050
Withers	19.925±3.543	33.848±7.053	57.794±12.030	459.523±61.167	911.971±106.700	1522.922±104.650	830±31.623	1476±30.984	4360±110	4.156	3.579	3.656
Scapula	16.683±2.499	29.020±5.442	44.677±8.841	475.6021±56.773	624.464±80.910	1166.082±116.810	772±23.476	1286±29.889	2858±66.966	3.389	4.441	3.690
Brachia	19.601±3.536	25.206±6.268	52.813±7.507	680.306±96.137	696.079±117.984	1624.216±78.242	856±15.776	1364±20.656	2440±160	2.800	3.495	3.149
Thorax	19.020±3.900	25.225±5.291	55.437±8.037	470.929±61.390	714.606±59.546	1399.918±105.301	764±20.656	1698±23.944	4376±92.760	3.882	3.410	3.809
Lateral of forearm	19.727±3.361	37.723±9.588	52.334±10.616	681.032±74.897	769.628±133.908	1498.445±167.616	720±21.082	1208±21.499	4064±59.479	2.815	4.672	3.375
Medial of forearm	19.729±3.638	33.415±7.380	50.663±8.255	569.574±69.671	581.731±85.103	1272.893±99.051	678±14.757	1148±21.499	3602±79.134	3.348	5.432	3.828
Metacarpus	29.307±5.684	63.594±22.655	78.276±15.893	636.031±94.067	1125.042±91.652	2181.566±117.097	818±23.944	1490±31.358	5324±65.862	4.405	5.350	3.464
Back	21.913±6.331	38.761±8.852	75.463±17.011	948.520±73.735	1210.813±63.443	2137.316±193.404	1500±249.443	1710±28.674	5934±114.717	2.258	3.102	3.410
Costal region	21.299±4.725	22.458±4.482	59.738±13.051	671.627±79.807	825.890±58.782	1438.027±85.229	884±26.331	1364±26.331	3132±84.958	3.074	2.647	3.988
Waist	20.536±4.077	35.372±7.345	63.847±12.527	443.341±62.320	796.013±94.636	1070.887±52.479	856±26.331	1464±26.331	4214±88.969	4.427	4.255	5.627
Buttock	25.034±6.210	32.513±6.427	49.577±8.090	683.558±111.933	864.660±100.912	1551.509±88.742	1432±23.476	2102±27.406	5088±71.305	3.533	3.624	3.096
Thigh	17.263±2.928	35.843±8.074	36.919±9.242	657.721±62.790	903.122±98.930	1352.018±91.040	760±13.333	1070±23.570	4084±49.710	2.558	3.817	2.658
Abdomen	26.960±7.136	25.973±5.384	44.790±8.235	545.228±64.915	881.225±61.878	1192.921±85.710	1154±23.190	1564±36.271	3106±89.963	4.712	2.863	3.619
Lateral of crus	16.073±3.439	33.289±8.968	47.443±7.790	450.651±43.227	731.228±101.218	1731.467±127.219	804±26.331	1370±25.386	3616±99.688	3.444	4.354	2.667
Inguinal region	17.104±3.541	27.415±5.991	34.211±7.284	457.078±45.998	507.742±57.984	923.596±81.996	624±18.379	976±30.984	2190±54.365	3.607	5.123	3.572
Medial of crus	19.110±3.993	34.908±8.247	44.104±6.188	523.636±62.925	736.896±115.078	1538.386±122.311	720±18.856	1240±18.856	3194±98.905	3.521	4.523	2.787
Metatarsus	24.355±5.684	30.194±10.061	64.585±10.767	389.956±59.511	793.938±71.907	1411.436±548.371	938±22.010	1318±40.497	4802±17.999	5.879	3.664	4.376
Axilla	17.714±4.025	30.201±7.434	43.784±5.653	449.609±71.736	532.978±98.182	826.451±58.989	724±20.656	1170±23.570	2052±73.756	3.791	5.363	5.031

**Table 4:** Rank order of thickness of skin according to age.

NO.	Epidermis(μm)			Dermis(μm)			Skin(μm)		
	newborn	half-year-old	adult	newborn	half-year-old	adult	newborn	half-year-old	adult
1	metacarpus29.307	metacarpus63.594	metacarpus78.276	back948.520	back1210.813	metacarpus2181.566	cheek1538	dorsal neck2364	back5934
2	abdomen26.960	back38.761	back75.463	forehead890.758	metacarpus1125.042	back2137.316	back1500	ventral neck2276	metacarpus5324
3	buttock25.034	lateral of forearm37.723	metatarsus64.585	cheek715.009	dorsal neck1117.065	dorsal neck2003.075	forehead1442	cheek2264	buttock5088
4	metatarsus24.355	thigh35.843	waist63.847	buttock683.558	forehead1035.519	ventral neck1969.327	buttock1432	forehead2188	metatarsus4802
5	forehead23.584	waist35.372	costal region59.738	lateral of forearm681.032	withers911.972	lateral neck1854.609	ventral neck1306	lateral neck2116	cheek4640
6	dorsal neck22.564	medial of crus34.908	withers57.794	brachia680.306	thigh903.122	forehead1805.675	dorsal neck1238	buttock2102	dorsal neck4396
7	back21.913	dorsal neck34.372	lateral neck57.781	costal region671.627	ventral neck897.889	lateral of crus1731.467	lateral neck1226	back1710	thorax4376
8	lateral neck21.718	withers33.848	thorax55.437	lateral neck668.446	abdomen881.225	brachia1624.216	abdomen1154	thorax1698	withers4360
9	costal region21.299	medial of forearm33.415	brachia52.813	thigh657.721	buttock864.660	buttock1551.509	metatarsus938	abdomen1564	ventral neck4294
10	waist20.536	lateral of crus33.289	lateral of forearm52.334	dorsal neck651.727	costal region825.890	medial of crus1538.386	costal region884	metacarpus1490	waist4214
11	ventral neck19.958	buttock32.513	medial of forearm50.663	metacarpus636.031	waist796.013	withers1522.922	brachia856	withers1476	thigh4084
12	withers19.925	lateral neck30.406	buttock49.577	ventral neck586.190	metatarsus793.938	lateral of forearm1498.445	waist856	waist1464	lateral of forearm4064
13	medial of forearm19.729	axilla30.201	lateral of crus47.443	medial of forearm569.574	lateral of forearm769.628	costal region1438.027	withers830	lateral of crus1370	lateral neck3946
14	lateral of forearm19.727	metatarsus30.194	forehead46.248	abdomen545.228	medial of crus736.896	cheek1428.367	metacarpus818	brachia1364	lateral of crus3616
15	brachia19.601	cheek29.050	abdomen44.790	medial of crus523.636	lateral of crus731.228	metatarsus1411.436	lateral of crus804	costal region1364	medial of forearm3602
16	medial of crus19.110	scapula29.020	scapula44.677	scapula475.602	cheek721.014	thorax1399.918	scapula772	metatarsus1318	medial of crus3194
17	Thorax19.020	forehead28.259	dorsal neck44.296	thorax470.929	thorax714.606	thigh1352.018	thorax764	scapula1286	costal region3132
18	axilla17.714	inguinal region27.415	medial of crus44.104	withers459.523	brachia696.079	medial of forearm1272.893	thigh760	medial of crus1240	abdomen3106
19	thigh17.263	ventral neck26.830	axilla43.784	inguinal region457.078	lateral neck687.223	abdomen1192.921	axilla724	lateral of forearm1208	forehead2910
20	inguinal region17.104	abdomen25.973	ventral neck41.214	lateral of crus450.651	scapula624.464	scapula1166.082	lateral of forearm720	axilla1170	scapula2858
21	cheek16.714	thorax25.225	cheek39.114	axilla449.609	medial of forearm581.731	waist1070.887	medial of crus720	medial of forearm1148	brachia2440
22	scapula16.683	brachia25.206	thigh36.919	waist443.341	axilla532.978	inguinal region923.596	medial of forearm678	thigh1070	inguinal region2190
23	lateral of crus16.073	costal region22.458	inguinal region34.211	metatarsus389.956	inguinal region507.742	axilla826.451	inguinal region624	inguinal region976	axilla2052

In the half-year-old group, the skin thickness ranged from 976 to 2364 μm; the inguinal region was thinnest (976 μm), and the cheek, ventral neck and dorsal neck were the thickest (2264–2364 μm) (Tables 3 and 4). The thickness of the epidermis varied from 22.458 to 63.594 μm. The thickness in the costal region, brachia, thorax and abdomen ranged from 22.458 to 25.973 μm. The metatarsus, buttock and dorsal neck were relatively thick (30.194–34.372 μm), and the metacarpus was thickest (63.594 μm) (Tables 3 and 4). The thickness of the dermis varied from 507.742 to 1210.813 μm. The thickness in the inguinal region, axilla and medial region of the forearm ranged from 507.742 to 581.731 μm. The lateral neck, cheek, costal region and thigh were relatively thick (687.223–903.122 μm), and the back was thickest (1210.813 μm) (Tables 3 and 4).

In the adult group, the skin thickness ranged from 2052 to 5934 μm; the axilla and inguinal regions were thinnest (2052 μm, 2190 μm), and the back and metacarpus were thickest (5934 μm, 5324 μm) (Tables 3 and 4). The thickness of the epidermis varied from 34.211 to 78.276 μm, and the thickness in the inguinal region, thigh, cheek and axilla ranged from 34.211 to 43.784 μm. The buttock, thorax, withers and costal region were relatively thick (49.577–59.738 μm); the metacarpus was thickest (78.276 μm) (Tables 3 and 4). The thickness of the dermis varied from 826.451 to 2181.566 μm, and the thickness in the axilla, inguinal region and waist ranged from 826.451 to 1070.887 μm. The abdomen, thorax, cheek and forehead were relatively thick (1192.921–1805.675 μm), and the back and metacarpus were the thickest (2137.316 μm, 2181.566 μm) (Tables 3 and 4).

The epidermis accounted for 2.258–5.879% of the entire skin in the newborn group, 2.647–5.432% in the half-year-old group, and 2.05–5.627% in the adult group (Table 3).

The thicknesses of the epidermis and dermis increased with age from newborn to adult. The age-related thickness changes differed significantly in the newborn, half-year-old and adult groups (Table 5). The differences in thicknesses of both the dermis and the skin were statistically significant among the three age groups (*P*<0.05), whereas the thicknesses of the epidermis varied among the three groups. In the thorax, back, costal region and abdomen, the thickness of the epidermis showed no significant difference between the newborn and half-year-old group (*P*>0.05) but was significantly different compared to the adult group (*P*<0.05).

**Table 5:** Age differences of thickness of epidermis, dermis and skin.

Region	Epidermis(μm)			Dermis(μm)			Skin(μm)		
	newborn	half-year-old	adult	newborn	half-year-old	Adult	newborn	half-year-old	adult
Forehead	23.584^c^	28.259^b^	46.248^a^	890.758^c^	1035.520^b^	1805.675^a^	1442^c^	2188^b^	2910^a^
Cheek	16.714^c^	29.049^b^	39.114^a^	715.009^c^	721.014^b^	1428.367^a^	1538^c^	2264^b^	4640^a^
Dorsal neck	22.564^c^	34.372^b^	44.296^a^	651.727^c^	1117.065^b^	2003.075^a^	1238^c^	2364^b^	4396^a^
Lateral neck	21.718c	30.406^b^	57.781^a^	668.446^c^	687.223^b^	1854.609^a^	1226^c^	2116^b^	3946^a^
Ventral neck	19.958^c^	26.830^b^	41.214^a^	586.189^c^	897.889^b^	1969.327^a^	1306^c^	2276^b^	4294^a^
Withers	19.925^c^	33.848^b^	57.794^a^	459.523^c^	911.972^b^	1522.922^a^	830^c^	1476^b^	4360^a^
Scapula	16.683^c^	29.020^b^	44.677^a^	475.602^c^	624.464^b^	1166.082^a^	772^c^	1286^b^	2858^a^
Brachia	19.601^c^	25.206^b^	52.813^a^	680.306^c^	696.079^b^	1624.216^a^	856^c^	1364^b^	2440^a^
Thorax	19.020^b^	25.225^b^	55.437^a^	470.929^c^	714.606^b^	1399.918^a^	764^c^	1698^b^	4376^a^
Lateral of forearm	19.727^c^	37.723^b^	52.334^a^	681.032^c^	769.628^b^	1498.445^a^	720^c^	1208^b^	4064^a^
Medial of forearm	19.729^c^	33.415^b^	50.663^a^	569.574^c^	581.731^b^	1272.893^a^	678^c^	1148^b^	3602^a^
Metacarpus	29.307^c^	63.594^b^	78.276^a^	636.031^c^	1125.042^b^	2181.566^a^	818^c^	1490^b^	5324^a^
Back	21.913^b^	38.761^b^	75.463^a^	948.520^c^	1210.813^b^	2137.316^a^	1500^c^	1710^b^	5934^a^
Costal region	21.299^b^	22.458^b^	59.738^a^	671.627^c^	825.890^b^	1438.027^a^	884^c^	1364^b^	3132^a^
Waist	20.536^c^	35.372^b^	63.847^a^	443.341^c^	796.013^b^	1070.887^a^	856^c^	1464^b^	4214^a^
Buttock	25.034^c^	32.513^b^	49.577^a^	683.558^c^	864.660^b^	1551.509^a^	1432^c^	2102^b^	5088^a^
Thigh	17.263^c^	35.843^b^	36.919^a^	657.721^c^	903.122^b^	1352.018^a^	760^c^	1070^b^	4084^a^
Abdomen	26.960^b^	25.973^b^	44.790^a^	545.228^c^	881.225^b^	1192.921^a^	1154^c^	1564^b^	3106^a^
Lateral of crus	16.073^c^	33.289^b^	47.443^a^	450.651^c^	731.228^b^	1731.467^a^	804^c^	1370^b^	3616^a^
Inguinal region	17.104^c^	27.415^b^	34.211^a^	457.078^c^	507.742^b^	923.596^a^	624^c^	976^b^	2190^a^
Medial of crus	19.110^c^	34.908^b^	44.104^a^	523.636^c^	736.896^b^	1538.386^a^	720^c^	1240^b^	3194^a^
Metatarsus	24.355^c^	30.194^b^	64.585^a^	389.956^c^	793.938^b^	1411.436^a^	938^c^	1318^b^	4802^a^
Axilla	17.714^c^	30.201^b^	43.784^a^	449.609^c^	532.978^b^	826.451^a^	724^c^	1170^b^	2052^a^

Different letters represent that the difference was significant in the same region (*p*<0.05), the same letter represents that the difference was no significant in the same region (*p*>0.05

**Fig 6:**
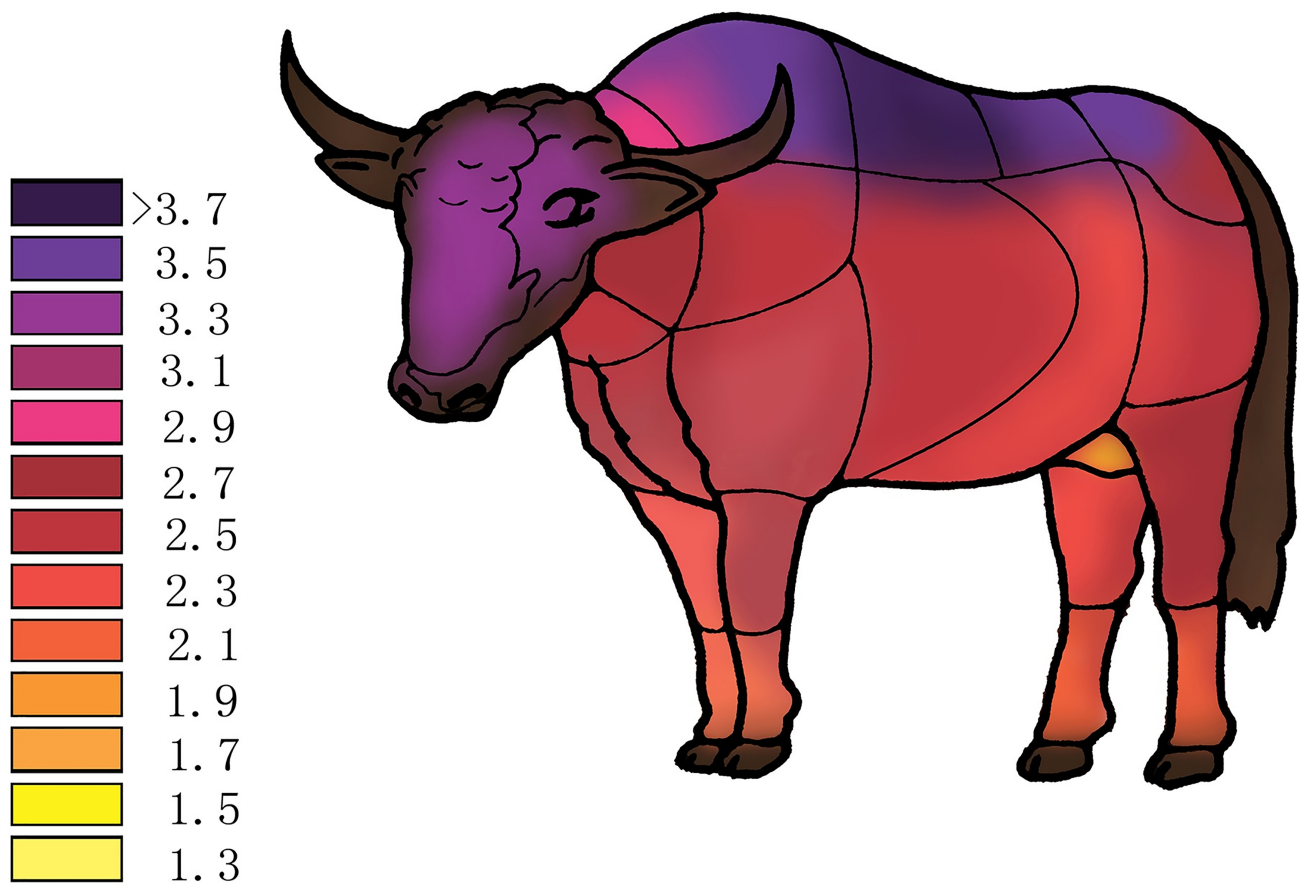
Schema graphs showing thickness change of skin in adult yak (unit: mm). Different colors show different thickness over body regions.

A schema graph was provided to show the changes in adult yak according to the data we obtained (Fig 6).

### Expression of HSP27 in skin during hair cycle

**Fig 7:**
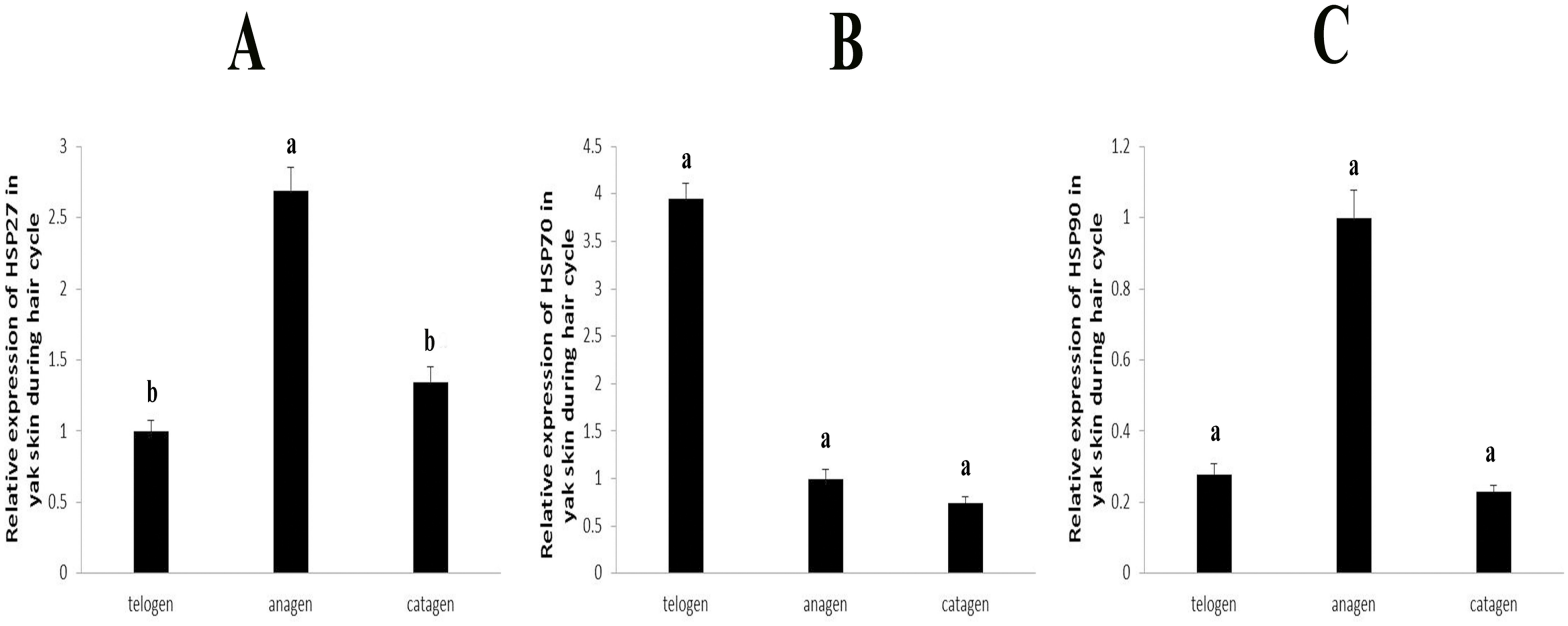
The HSPs gene expressions in skin of yak during hair cycle. A: HSP27 gene expression in skin. B: HSP70 gene expression in skin. C: HSP90 gene expression in skin. Different letters represent that the difference was significant (*p*<0.05), the same letter represents that the difference was no significant (*p*>0.05).

**Fig 8:**
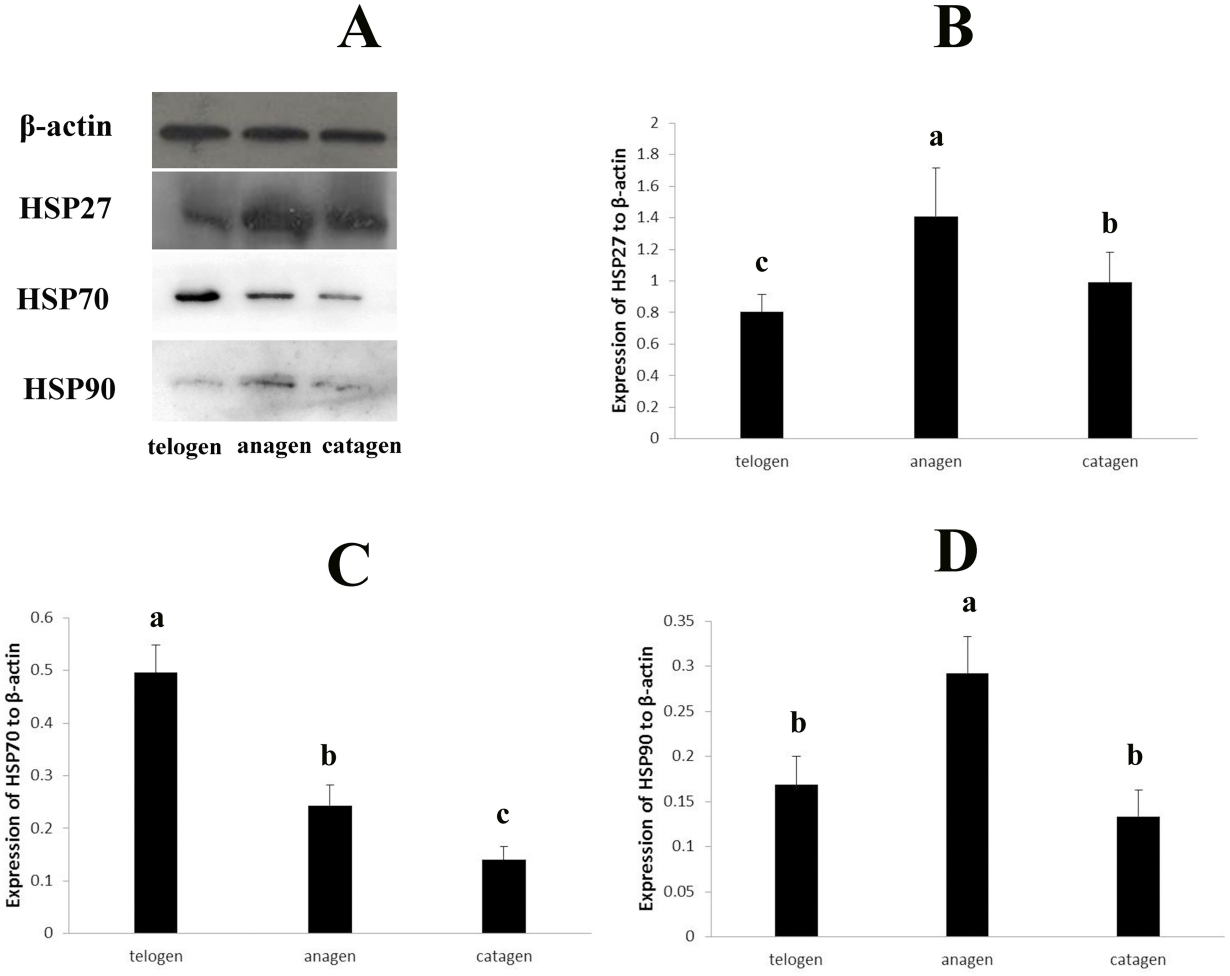
Detection of HSP27, HSP70 and HSP90 expression in skin of yak during hair cycle by Western-blot. Different letters represent that the difference was significant (*p*<0.05), the same letter represents that the difference was no significant (*p*>0.05).

**Fig 9:**
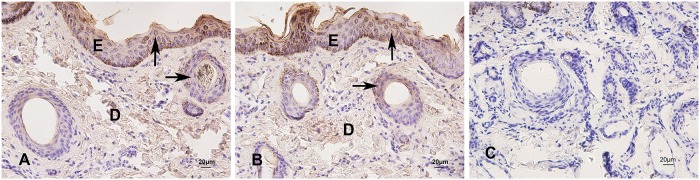
Immunohistochemical staining of HSP27 in skin of yak during hair cycle. A, B: HSP27 expressed in the epidermis and the outer root sheath of secondary follicle. C. negative control. Arrows show the immunostained products as brown deposits. ×400.

A fluctuation in the relative expression levels of HSP27 mRNA during the hair cycle was shown in Fig 7A. In the hair cycle, the highest level of HSP27 mRNA expression was found during the anagen stage, whereas the lowest expression level was found in the telogen stage. The expression level in the catagen stage was between the anagen and telogen stages. The expression level showed significant difference between anagen and telogen stages (*P*<0.05) as well as between anagen and catagen stages (*P*<0.05), but there was no difference between telogen and catagen stages (*P*>0.05). A similar expression pattern was observed for the HSP27 protein using western blot analysis (Fig 8A and 8B). The highest expression level was seen in the anagen stage, followed by the catagen stage, with the lowest level seen in the telogen stage. The result showed that the HSP27 protein expression levels were significantly different among hair cycle (*P*<0.05). HSP27 was mainly expressed in the outer root sheath of the secondary follicle during the hair cycle, also expressed in epidermis and sebaceous gland in the skin of yak (Fig 9).

### Expression of HSP70 in skin during hair cycle

**Fig 10:**
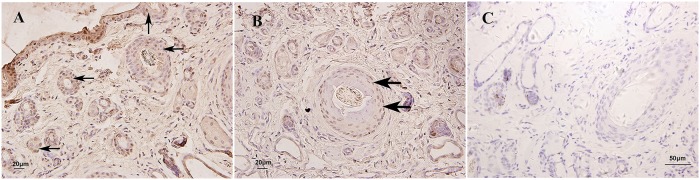
Immunohistochemical staining of HSP70 in skin of yak during hair cycle. A, B: HSP70 expressed in the epidermis, sebaceous gland, sweat gland and the outer root sheath of hair follicle. C. negative control. Arrows show the immunostained products as brown deposits. ×400.

A fluctuation in the relative expression levels of HSP70 mRNA during the hair cycle was shown in Fig 7B. In the hair cycle, the highest level of HSP70 mRNA expression was found during the telogen stage, whereas the lowest expression level was found in the catagen stage. The expression level in the anagen stage was between the telogen and catagen stages. There was no significant difference among three stages (*P*>0.05). A similar expression pattern was observed for the HSP70 protein using western blot analysis (Fig 8A and 8C). The highest expression level was seen in the telogen stage, followed by the anagen stage, with the lowest level seen in the catagen stage. However, there was significant difference of HSP70 protein expression levels among three stages (*P*<0.05). HSP70 protein expression was observed in the epidermis, sebaceous gland, sweat gland and outer root sheath of hair follicle in the skin (Fig 10).

### Expression of HSP90 in skin during hair cycle

**Fig 11:**
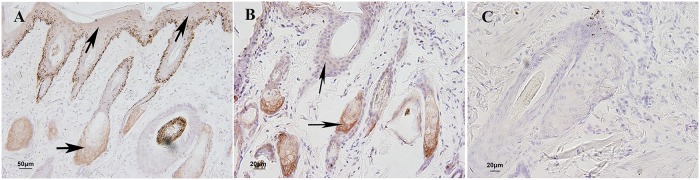
Immunohistochemical staining of HSP90 in skin of yak during hair cycle. A, B: HSP90 expressed in the epidermis, sebaceous gland and hair root sheath. C. negative control. Arrows show the immunostained products as brown deposits. ×400.

A fluctuation in the relative expression levels of HSP90 mRNA during the hair cycle was shown in Fig 7C. In the hair cycle, the highest level of HSP90 mRNA expression was found during the anagen stage, whereas the lowest expression level was found in the catagen stage. The expression level in the telogen stage was between the anagen and catagen stages. There was no significant difference among three stages (*P*>0.05). A similar expression pattern was observed for the HSP90 protein using western blot analysis (Fig 8A and 8D). The highest expression level was seen in the anagen stage, followed by the telogen stage, with the lowest level seen in the catagen stage. The expression levels of HSP90 protein showed significant difference between anagen and telogen stages (*P*<0.05) as well as between anagen and catagen stages (*P*<0.05), but there was no difference between telogen and catagen stages (*P*>0.05). HSP90 protein expression was observed in the epidermis, sebaceous gland and hair root sheath in the skin (Fig 11).

### Expression of CGI-58 (ABHD5) in skin during hair cycle

**Fig 12:**
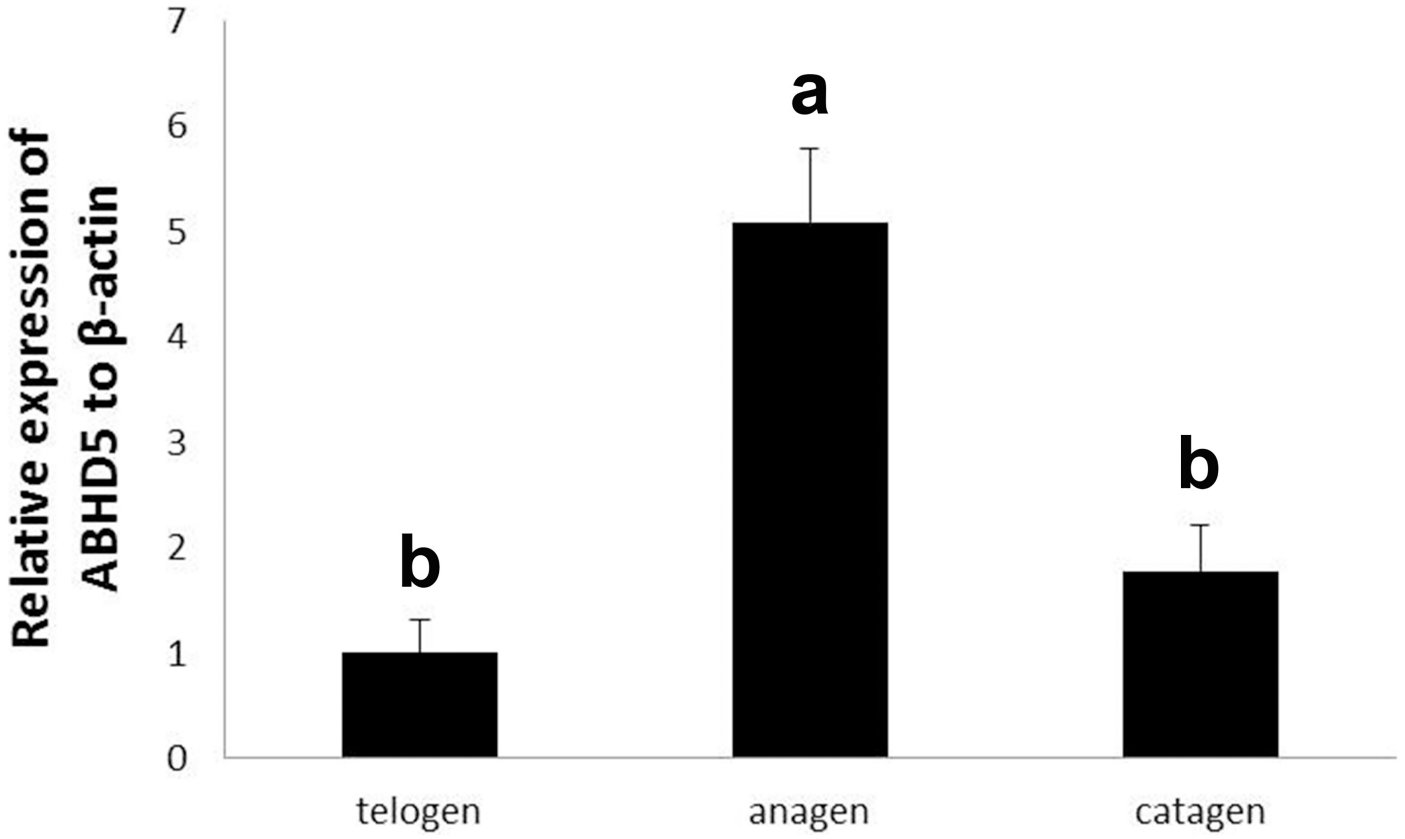
The CGI-58 gene expressions in skin of yak during hair cycle.

A fluctuation in the relative expression levels of CGI-58 mRNA during the hair cycle was shown in Fig 12. In the hair cycle, the highest level of CGI-58 mRNA expression was found during the anagen stage, whereas the lowest expression level was found in the telogen stage. The expression level in the catagen stage was between the anagen and telogen stages. The expression level showed significant difference between anagen and telogen stages (*P*<0.05) as well as between anagen and catagen stages (*P*<0.05), but there was no difference between telogen and catagen stages (*P*>0.05).

### Expression of KDF1 in skin during hair cycle

**Fig 13:**
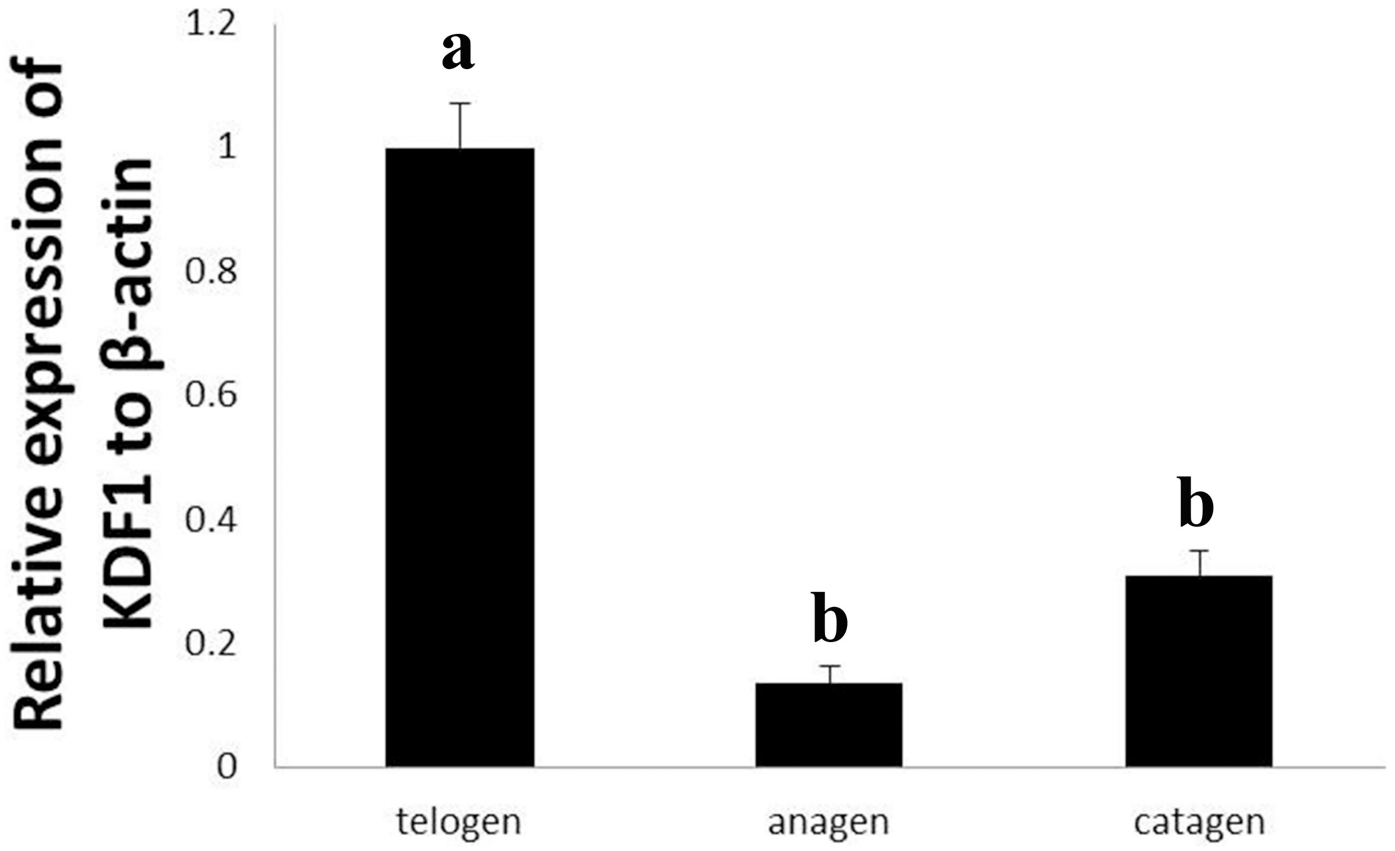
The KDF1 gene expressions in skin of yak during hair cycle.

A fluctuation in the relative expression levels of KDF1 mRNA during the hair cycle was shown in Fig 13. In the hair cycle, the highest level of KDF1 mRNA expression was found during the telogen stage, whereas the lowest expression level was found in the anagen stage. The expression level in the catagen stage was between the telogen and anagen stages. The expression level showed significant difference between telogen and anagen stages (*P*<0.05) as well as between telogen and catagen stages (*P*<0.05), but there was no difference between catagen and anagen stages (*P*>0.05).

## Discussion

### Modified method

To observe the histological structure of the hair follicle clearly, we used the Sacpic stain method, which was well suited for the visual assessment of follicle activity because it accentuates the inner root sheath. Tissue types were clearly defined. Results: nuclei, dark blue; keratin, yellow; collagen, blue; inner root sheath, bright red; outer root sheath, pale green; smooth muscle, green [42].

The shrinkage effect is the most important problem to prevent when measuring skin thickness. As soon as we harvested the skin, we fixed it to a paperboard by putting pins into the four corners and stored it in 4% paraformaldehyde solution. We believe that this procedure could prevent most of the shrinkage effect in the transverse plane.

### Histologic characteristics of skin structure

We confirmed that yak skin is composed of two layers: the epidermis and dermis. The total epidermis of the hairy skin consisted of the stratum corneum and the viable epidermis (stratum basale, stratum spinosum, stratum granulosum). The pelt was composed of compound hair follicles, which produced a primary and some secondary hair follicles. Associated with each primary follicle, there was an arrector pili muscle, a multilobular sebaceous gland, and a coiled tubular sweat gland. The difference between the primary and secondary follicles was that the primary follicles had their own sweat glands but that the secondary follicles did not bear sweat glands [43]. We confirmed that the yak hair follicle group consisted of one primary follicle and several secondary follicles, which was similar to that found in ferrets [19] but different from Iranian sheep breeds [43], Camelus dromedaries [44], Australian cashmere goat [45], llama [17] and sheep [18]. The sweat glands in yaks are not well developed. Sweat secretion does not occur readily, thereby reducing the heat radiation surface. This appears to force the animal to retain heat in the body and helps increase its tolerance to cold [46].

### Age-rated thickness change

In this study, we first measured the skin thickness of different ages and in different regions of yak. Skin thickness varied in different regions of the body surface in yak. The thickest-haired skin was present on the cheek, forehead, dorsal neck and ventral neck in the newborn and half-year-old groups. The thinnest part in the newborn and half-year-old groups was the inguinal region. The thickest haired skin was present on the back, followed by the metacarpus and buttocks in the adult group. The thinnest haired skin in the adult group was the inguinal region and the axilla. In a group of newborn and half-year-old yak, the area around the head, cheek and neck were thicker than other parts of the body surface, which was similar to llama [17]. The thickest location was the back in the adults, which corresponds to equine skin [21] and had previously reported in yak [46], perhaps because the back is the part of the body that is most exposed to wind, rain and snow. Yak skin thickness decreased dorsally to ventrally on the trunk. This pattern of skin thickness change was typical of most domestic large animals [47]. In yak, the skin on the lateral surface was thicker than the skin on the medial surface in the limbs.

The total thickness increased with age. Previous researchers noted that sunlight appears to have a considerable effect on the thickness and physical properties of skin [48,49]. Collagen was a major component of skin, and the age-related changes in thickness correlate well with skin collagen content [50]. The significant change in the epidermis in adults was obvious. The corneum layer increased with age, which was similar to the reports of Mugale [51].

Knowledge of skin thickness in yak may be useful in harvesting full- or split-thickness skin grafts to produce leather. Moreover, these results were useful for studying the relation between age-related thickness changes of skin and the living environment.

### Expression of HSPs in skin during hair cycle

This study reported for the first time the expression patterns of HSP27, HSP70 and HSP90 in skin during the hair cycle in yak. The HSP27 protein expression in the epidermis suggested that this protein may be useful for keratinocyte cell growth and regeneration, which concurs with previous studies [28–32]. In human epidermal keratinocytes, the expression of HSP27 was closely related to differentiation both in vitro and in situ [52]. HSP27 and p38-MAPK serve essential functions in the maintenance of the epidermal structure, and HSP27 was associated with keratinocyte differentiation [53]. Moreover, the expression of HSP27 in the epidermis showed that HSP27 may be the target for immune response and could protect against pathogens [25,26,54–56].

The highest expression of HSP27 during anagen and its weak expression in catagen and telogen agreed with the results in the mouse model [57]. The expression pattern suggested HSP27 may be involved in the hair follicle cellular cycle [58]. HSP27 expression in the hair cycle could be related to both keratinocyte differentiation and apoptosis in the hair follicle. HSP27 may promote and prolong anagen by protecting hair follicle keratinocytes against apoptosis [59]. Numerous studies have shown that HSP27 inactivates the caspase cascade by binding with caspase-3 and cytochrome C released from mitochondria and that it thus prevents apoptosis [60–62]. The weak expression of HSP27 in catagen and telogen may be followed by the process of terminal differentiation and apoptosis of the keratinocyte. HSP27 could mediate this process by inducing some growth factors, such as FGF. In general, the expression pattern suggested that HSP27 expression may correlate with the level of differentiation of the keratinocytes and the level of keratinization of the outer root sheath.

The predominant expression in epidermis of HSP70 and HSP90 protein as well as HSP27 suggested that they may also involve in keratinocyte cell growth and differentiation. HSP70 played an important role in cell apoptosis. In testis, ablation of HSP70 isoform resulted in germ cell apoptosis [63]. HSP90 was known as a molecular chaperone and had other functions. HSP90 can control cell proliferation by stabilizing the client proteins N-RAS and B-RAF [64,65]. The HSP90 protein was weakly expressed in all hair cycle stages compared with HSP27 and HSP70. This result agreed with Wilson’s study [66]. Although HSP90 was abundantly expressed in other tissues, it was not largely present in skin [39]. The mRNA expression levels of HSP70 and HSP90 showed no difference among three stages, but both of the two protein expression levels showed significant difference in all three stages, we believed that it related to the process of transcription regulation and would study deeply on this part. Otherwise, it may also be related to the interaction between HSPs proteins.

This study had demonstrated the expression pattern of HSP27, HSP70 and HSP90 in yak skin during hair cycle. All of three HSP proteins were involved in the hair follicle cellular cycle and may related with cell apoptosis. However, the different expression patterns suggested that the function of each HSP protein was various. Our further research at this moment may give a definite mechanism next.

### Expression of CGI-58 and KDF1 in skin during hair cycle

We also detected the mRNA expression levels of CGI-58 and KDF1 in skin during the hair cycle in yak. CGI-58 showed the same expression pattern with HSP27 in mRNA level. The expression of CGI-58 mRNA in the anagen stage was the highest, followed by the catagen stage, and the expression in the telogen stage was the lowest. CGI-58 mRNA expression was up-regulated concomitantly with both epidermal stratification and keratinocyte differentiation [67]. The same pattern in skin during the hair cycle in yak suggested both CGI-58 and HSP27 were involved in keratinocyte differentiation in hair follicles. The expression of KDF1 mRNA was contrary to CGI-58.

The highest level was in the telogen stage, followed by the catagen stage, and the expression in the anagen stage was the lowest. KDF1 was expressed in epidermal progenitor cells and the progeny where it curbed proliferation as well as blocked proliferation and promoted differentiation [68]. The cycle-dependent expression of KDF1 suggested it may be relate to the proliferation state of hair follicle keratinocytes.
